# Supplementary material for: OncotreeVIS—an interactive graphical user interface for visualizing mutation tree cohorts
Source: Bioinform Adv. 2025 Oct 9;5(1):vbaf247. doi: 10.1093/bioadv/vbaf247 (PMC12596139; doi:10.1093/bioadv/vbaf247)
Supplement: vbaf247_Supplementary_Data [file vbaf247_supplementary_data.pdf]

# OncotreeVIS – an interactive graphical user interface for visualizing mutation tree cohorts

**Monica-Andreea Baciu-Drăgan[1,2] and Niko Beerenwinkel[1,2,*]**

[1] Department of Biosystems Science and Engineering, ETH Zürich, Schanzenstrasse 44, 4056, Basel, Switzerland
[2] SIB Swiss Institute of Bioinformatics, Schanzenstrasse 44, 4056, Basel, Switzerland

[*]Corresponding author. E-mail: niko.beerenwinkel@bsse.ethz.ch

# Supplementary information

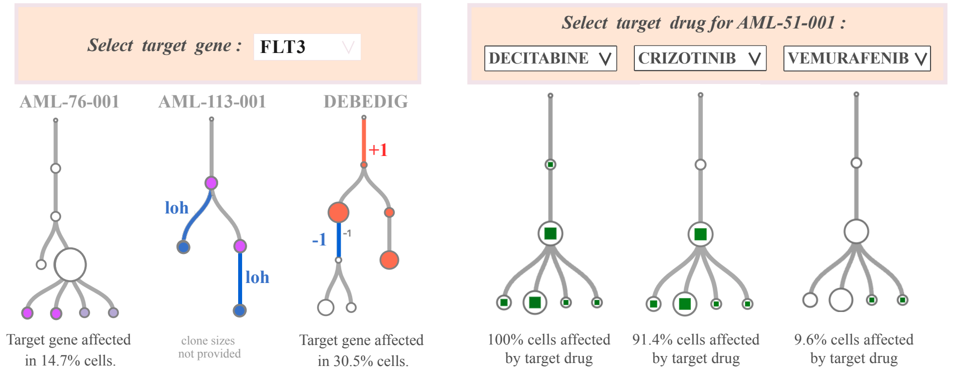


A. Highlighting subclones that present a mutation in the target gene, or interact with a target drug.

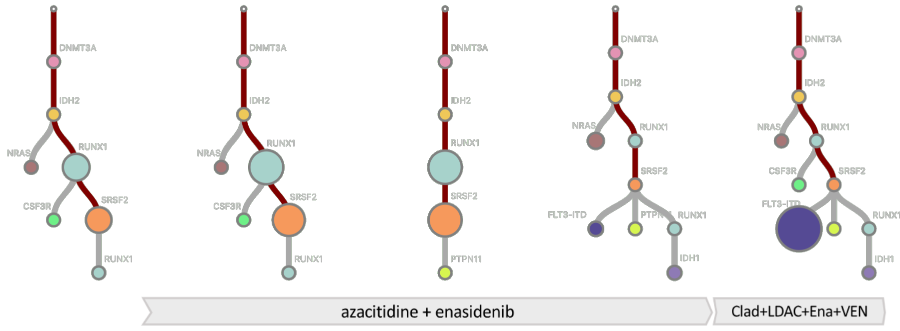


B. Longitudinal AML samples for patient AML-99 from Morita et al. 2020.

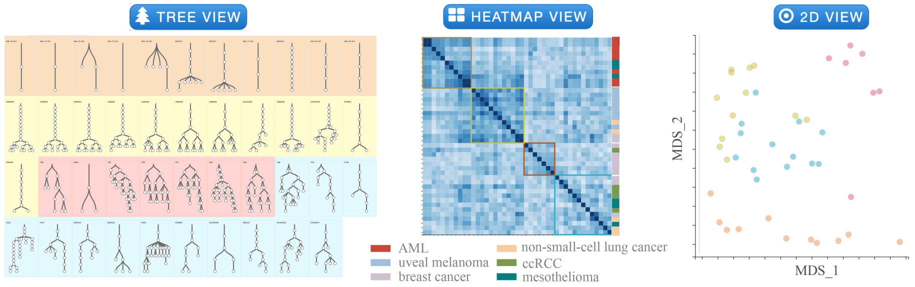


C. Visualization of different modes of spatial tumor evolution.

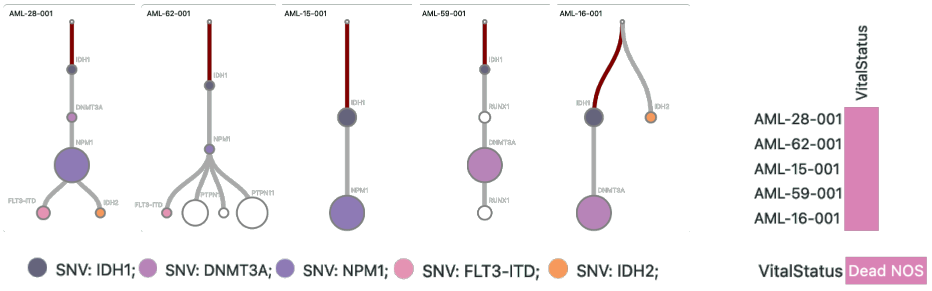


D. Details of an AML mutation tree cluster gouverned by primary mutation IDH.

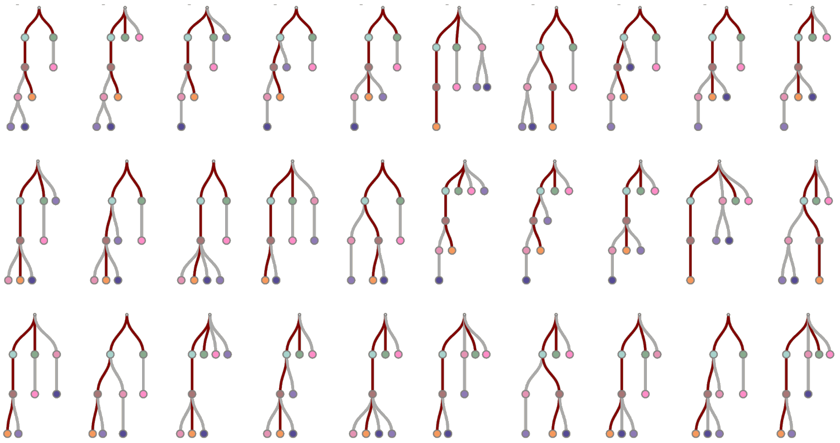

E. Alternative phylogenies inferred by CONIPHER for sample sample CRUK0100.

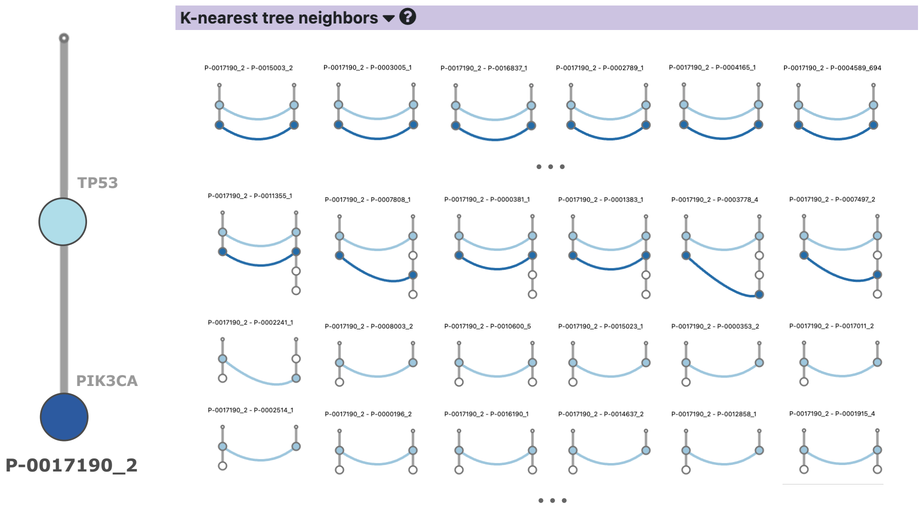


F. K-nearest tree neighbors for sample P-0017190_2 from Razavi et al., 2018

**Supplementary Figure 1: OncotreeVIS data views for different use cases: (A) Left:** highlighting subclones that present a mutation in gene FLT3 for samples AML-76-001 (Morita *et al.*, 2020), AML-113-001 (Sollier *et al.*, 2023) and DEBEDIG (Wegmann *et al.*, 2024). Point mutations are shown with violet, CN amplification events in red, and CN deletions and loss of heterozygosity in blue. For AML-113-001, the arising of a mutation in FLT3 is subsequently followed by loss of heterozygosity in the same gene. Also, in DEBEDIG mutation events (i.e., amplifications and deletions) affecting the same gene succeed one another over time. **Right:** highlighting subclones affected by three different target drugs according to the drug-gene interaction database (Cannon *et al.*, 2024) for sample AML-51-001 (Morita *et al.*, 2020). We consider that a target drug can have therapeutic impact on a subclone if there is a known database interaction between the drug and at least one of the mutated genes in the subclone. Here, DECITABINE affects all the subclones, CRIZOTINIB affects 91.4% of the cells (all the subclones except for the clone following directly the root), and VEMURAFENIB affects 9.6% of the cells (only the two small subclones disposed at the leaves of the tree). The full list of gene-drug interactions can be found in the "Top DGIdb drugs associated with target gene" section of the interface, available upon clicking each individual tree. **(B)** Five longitudinal AML samples for patient AML-99 (Morita *et al.*, 2020) obtained before and during treatment, including treatment change. The tumor evolution shown at different timepoints reveals the underlying process of therapeutic resistance, namely the emergence of IDH1/FLT3/NRAS clones during IDH2 inhibitor-containing therapy; **(C)** Cohort overview, heatmap, and 2D visualization of 43 tumor mutation trees from 6 cancer types and different modes of spatial tumor evolution selected in Noble *et al.* (2022), clustered with oncotree2vec (Baciu-Drăgan and Beerenwinkel, 2024). The clusters are indicated by different colors and correspond to four modes of evolution: linear (orange cluster), punctuated (yellow cluster), branching (red cluster) and linear-to-branched evolution (blue cluster). **(D)** Details of an AML mutation tree cluster output by oncotree2vec after clustering the Morita *et al.* (2020) tree cohort, governed by primary mutation IDH2. **(E)** Alternative phylogenies inferred by CONIPHER mutation tree inference algorithm (Grigoriadis *et al.*, 2024) for sample CRUK0100. Conserved trajectories between the alternative trees are highlighted in dark red. **(F)** Selection of mutation trees matching target sample P-0017190_2 from Razavi *et al.* (2018). The panel shows pairs of matching trees (where the left tree always corresponds to the target sample) and the corresponding matching subclones. In 79 of the cases the matching corresponds to the highly conserved trajectory TP53 → PIK3CA reported in Luo *et al.* (2023) for the cohort of breast cancer point mutation trees from Razavi *et al.* (2018). Using a similar approach starting from P-0010235_43, 66 matching trees were found in the same cohort for the evolutionary trajectory PIK3CA → TP53, which is assigned a lower probability according to Luo *et al.* (2023). This example shows how oncotreeVIS can be used to interpret the results of algorithms that identify conserved evolutionary trajectories.

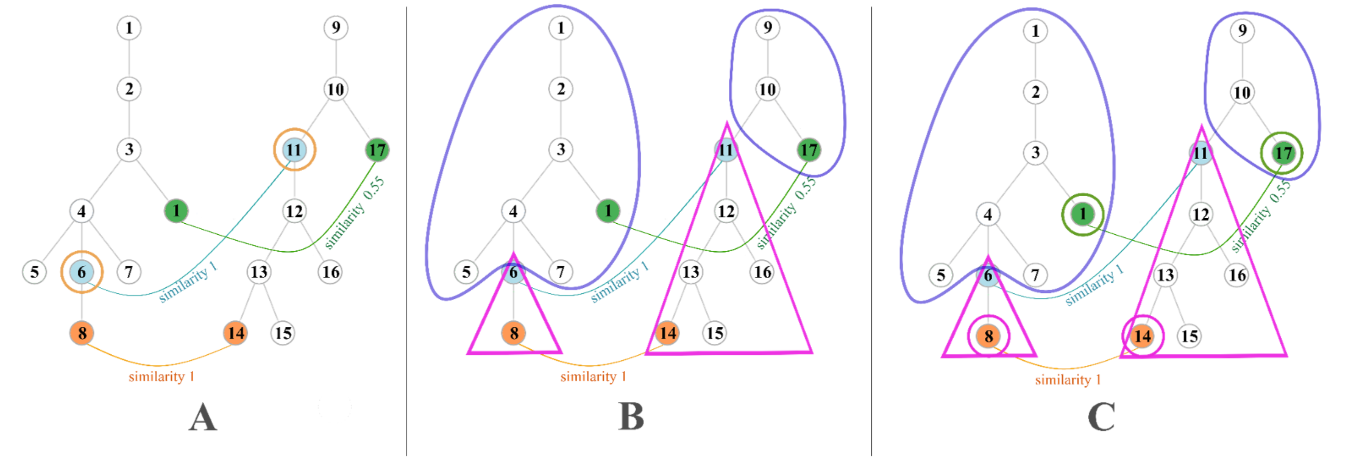


**Supplementary Figure 2: Example of how the proposed heuristic for the maximum matching problem with ordering constraints works** for two trees with three matching nodes with similarities 1, 1 and 0.55. Each node has an unique id to make it easier to be referred. The nodes sharing the same color have matching label sets with a high similarity (the similarity score is specified along the corresponding link). White colored nodes are not matching any other node. The node matching heuristic starts from the best matching node between the two trees (highest similarity, lowest depth). Starting from the matching node pair, the same greedy matching process is applied recursively to the children trees and to the remaining upper trees, until all the nodes are considered. The algorithm of the recursive matching function in provided in Algorithm 1. **(A)** First, nodes with ids 6 and 11 are matched (maximum similarity and lowest depth). **(B)** The nodes from the child trees (the pink triangles) and from the outer trees (the blue triangles) are matched recurrently. **(C)** Inside the recursion, nodes with ids 8 and 14, and 1 and 17 respectively, are matched.

**Algorithm 1** A heuristic for the maximum matching with ordering constraints.

```
input: data structures for two labeled trees, tree_1 and tree_2.
procedure find_matching_nodes(tree_1, tree_2) :
node_tree_1, node_tree_2 = get_best_node_match(tree_1, tree_2)

                                        ▷ Run the matching for the children subtrees.
if has_children(node_tree_1) and has_children(node_tree_2) then
    subtree_1 = get_child_subtree(tree_1, node_tree_1)
    subtree_2 = get_child_subtree(tree_2, node_tree_2)
    matches_children = find_matching_nodes (child_tree_1, child_tree_2)
end if

                                        ▷ Run the matching for the remaining upper tree.
upper_tree_1 = get_upper_tree(tree_1, node_tree_1)
upper_tree_2 = get_upper_tree(tree_2, node_tree_2)
upper_matches = find_matching_nodes(upper_tree_1, upper_tree_2)

best_match = tuple(node_tree_1, node_tree_2)

return best_match, *matches_children, *upper_matches
```

**Supplementary Table 1: Public mutation tree cohorts visualized by default in the oncotreeVIS web application.** The details of the tree cohort from Noble *et al.* (2022) are not reported because it is a mixture of 5 datasets. Abbreviations: single-cell (SC), multi-region (MR), whole-genome sequencing (WGS), whole-exome sequencing (WES)

| **Dataset** | **Description** | **Sequencing type** | **Mutation tree inference tool** |
|---|---|---|---|
| Morita *et al.* (2020) | 123 AML point mutation trees, latest sample for each patient (clustered).<br>The mutation trees were inferred from single-cell DNA sequencing data (single-time biopsies and longitudinal samples) from Morita *et al.* (2020) and clustered with oncotree2vec as described in (Baciu-Drăgan and Beerenwinkel, 2024). Thirteen clusters were found, ranging in size from 18 to 4 samples, governed by the primary gene mutation. | SC, panel | SCITE (Jahn *et al.*, 2016) |
| Noble *et al.* (2022) | 43 mutation trees with different modes of evolution, from 5 cancer types (clustered).<br>The trees were collected in Noble *et al.* (2022) from different publications, for six cancer types: acute myeloid leukemia (AML, 8 samples), clear cell renal cell carcinoma (ccRCC, 5 samples), mesothelioma (6 samples), breast cancer (11 samples), non-small cell lung cancer (5 samples) and uveal melanoma (8 samples). We used oncotree2vec to cluster the trees based on the tree structure (by ignoring the node labels) as described in (Baciu-Drăgan and Beerenwinkel, 2024). The resulting clusters correspond to four distinct modes of spatial tumor evolution: linear, punctuated, branching and linear-to-branched. | NA | NA |
| TRACERx (Jamal-Hanjani *et al.*, 2017) | 137 point mutation trees from 99 non-small cell lung cancer patients (clustered).<br>The mutation trees were inferred with REVOLVER (Caravagna *et al.*, 2018) from the TRACERx NSCLC non-small-cell lung cancer multi-region whole-exome sequencing data (Jamal-Hanjani *et al.*, 2017), using a maximum likelihood approach. For ten of the patients, the model outputted multiple plausible cell phylogenies with comparable likelihoods, and for the rest of the patients the most likely reconstructed tree was reported. The trees were clustered using oncotree2vec, as described in (Baciu-Drăgan and Beerenwinkel, 2024). As expected, the resulting clustering of the mutation trees in the embedding space reflects the different posterior distributions for the samples with alternative cell phylogenies, i.e., the trees which correspond to the same posterior distribution cluster together. | MS, WES | Jamal-Hanjani *et al.* (2017) |
| TRACERx421 (Al Bakir *et al.*, 2023) | 4,843 alternative phylogenies inferred for 126 non-small cell lung metastatic cancer patients. The cohort includes a combinatorial enumeration of all alternative cell phylogenies for each sample (up to 100 alternative trees per sample). The trees are grouped by sample. | MS, WES | CONIPHER (Grigoriadis *et al.*, 2024) |
| Razavi *et al.* (2018) | 1,214 breast cancer point mutation trees (1,152 patients) preprocessed following Christensen *et al.* (2020). | Bulk | SPRUCE (El-Kebir *et al.*, 2016) |
| Sollier *et al.* (2023) | 145 AML joint copy number and point mutation trees – same raw data as Morita *et al.* (2020). | SC, panel | COMPASS (Sollier *et al.*, 2023) |
| Wegmann *et al.* (2024) | 21 copy number trees from 16 patients. | SC, WGS | SCICoNE (Kuipers *et al.*, 2025) |

**Supplementary Table 2: Example of input data encoding the information about a tree cohort in JSON format.** Key values are highlighted in bold. Scripts for converting the output of different tree inference tools (see Suppl. Table 3) into the JSON format used by oncotreeVIS JSON are provided at https://github.com/cbg-ethz/oncotreevis.

| **Key** | **Data structure** |
|---|---|
| trees | Nested data structure representing a tree in JSON format, as used in D3.js and anytree (python) libraries. Each node has one or more child nodes (`node.children`), except for the leaves. In addition, each node has the following attributes: `node_id` (string/int, required), `matching_label` (required), `metadata` (dictionary, optional), `gene_events` (dictionary, optional), `is_neutral` (boolean, optional). The gene_events attribute has two predefined keys (”mutation” and ”CNA”), but any other key names can be used. The values for the ”CNA” event key are specifically interpreted as amplification or deletion amounts w.r.t. the neutral states. The first three letters of the event key are used in the visualization for displaying a summary for the gene events.<br><br>Example of JSON tree:<br>`"AML-03-001":{"tree":{"node_id":0,"matching_label":0,"children":`<br>`[{"node_id":407,"matching_label":14,"size_percent":0.228,`<br>`"gene_events":{"FLT3-ITD":{"mutation":""}},"children":[{"node_id":`<br>`408,"matching_label":5,"size_percent":0.772,"gene_events":{"NPM1":`<br>`{"mutation":"p.L287fs"}}}]}]},"metadata":{"Chemo":"No","Gender":`<br>`"Female","VitalStatus":"Dead","age":59,"Response":"CR"}}` |
| clusters | List of lists of tree ids (strings). Example:<br>`[['AML-55-001', 'AML-33-001', 'AML-57-001', 'AML-11-001'],`<br>`['AML-77-001'], ['AML-50-001', 'AML-102-001'], ... ]` |
| pairwise_tree_distances | List of dictionaries where the keys are the tree ids of the pair of trees (strings) and the values are the distance scores (float). Example:<br>`[{'sample_1': 'AML-73-001', 'sample_2': 'AML-22-001',`<br>`'similarity': 0.6072}, ... ]` |
| highlighted_genes | Styles used: color code or keywords ”bold”, ”italic”, ”underline”. Example:<br>`{"JAK2": "bold", "PTEN": "italic", "TP53": "#b4a7d6",`<br>`"FLT3-ITD": "lightsteelblue"}` |

**Supplementary Table 3: Output formats for the tumor mutation trees output by different tree inference tools visualized by default in the oncotreeVIS web application. In our code repository we provide scripts to convert all these data formats into the JSON input format used by oncotreeVIS.**

| Raw data / tree cohort | Tree inference tool | Output format |
|---|---|---|
| Morita *et al.* (2020) | SCITE (Jahn *et al.*, 2016) | Graphviz representation (Ellson *et al.*, 2002) of each individual tree. |
| Sollier *et al.* (2023) | COMPASS (Sollier *et al.*, 2023) | List of dictionaries per node, for each individual tree. |
| Wegmann *et al.* (2024) | SCICoNE (Kuipers *et al.*, 2025) | Graphviz representation (Ellson *et al.*, 2002) of each individual tree. |
| Razavi *et al.* (2018) | SPRUCE (El-Kebir *et al.*, 2016) | The first row of the file indicates how many alternative phylogenies were detected by the tree-building algorithm. Each alternative tumor phylogeny number X begins with a header: "# tree X". For each tree, each new row is a tree branch between two node labels.<br><br>Example:<br>### 11 trees<br># tree 1<br>2 1<br>8 3<br>21 4<br>1 5<br>. . . |
| TRACERx<br>(Jamal-Hanjani *et al.*, 2017) | Jamal-Hanjani *et al.* (2017) | *Idem.* |
| TRACERx421<br>(Al Bakir *et al.*, 2023) | CONIPHER (Grigoriadis *et al.*, 2024) | *Idem.* |
| Noble *et al.* (2022) | NA | CSV file with adjacency list in tabular format, for each individual tree.<br><br>Example:<br>Parent Identity Population<br>0 0 0<br>0 0a 0<br>0a 1 2.03<br>1 4 0.71<br>. . . |

**Supplementary Table 4: Implementation details.**

| Clickable elements | Action |
|---|---|
| | Zoom in tree cohort canvas. |
| | Zoom out tree cohort canvas. |
| | Reset zoom. |
| | Download camera-ready PDF figure. Large cohorts will be truncated – jsPDF limits the width/height to 14400px. |
| TREE VIEW | Shows mutation trees side by side, grouped by a given clustering (by default). Nodes correspond to clones of different sizes. Each node is labeled with the set of provided gene mutations (SNVs, CNAs, etc) acquired by the subclone (also displayed on the incoming edges). Matching subclones and conserved edges are highlighted. Neutral clones (if specified) are colored in light yellow. |
| **Sorting options:** | |
| | Default state: Shows trees grouped by given input clustering. |
| A Z | Shows all the trees sorted in alphabetical order. |
| | Shows all the trees in random order. |
| **Highlight matching subclones and trajectories:** | |
| | Default state: Matching subclones (i.e., nodes with the same `matching_label`) are indicated by colors and conserved edges (i.e., pairs of `matching_label` values for parent-child relations) are highlighted in each cluster. Each `matching_label` is assigned with a different color. For a better user experience, in clusters where more than 10 colors are needed, the matching label is also displayed on top of each node – if the length of the matching label string exceeds the size of the node circle, the value is displayed only when the node is hovered by the mouse pointer. The colors are consistent among the different subclones. If no clusters are provided (or a sorting option is selected), all the trees are seen as one big cluster. In this case the conserved edges are not computed. |
| | Node colors are removed. If clusters are provided, then the conserved edges remain highlighted. This option is not applicable when a sorting option is selected. |
| | Only the matching subclones are indicated by matching colors. |
| HEATMAP VIEW | Shows a heatmap visualization of the given pairwise distances between the mutation trees. |
| | Remove the highlighted clusters on the heatmap. |
| | Show the highlighted clusters on the heatmap. |
| 2D VIEW | Shows a 2D projection of the tree points based on a given tree pairwise distances, using Multidimensional scaling (MDS). |
| -- Select target gene event -- | Subclones affected by the selected target gene are highlighted with colors in the mutation tree below: red for CN amplification, blue for CN deletion and violet for any other mutation event. |
| -- Select target drug -- | Subclones with affected genes that have a theoretical interaction with the selected target drug (according to DGIdb) are indicated with green squares in the mutation tree below. Drugs are listed in descending order by the number of cells they affect. Only drug interactions with at least 3 citations are considered. |
| show cluster details | The following cluster details are displayed: a summary of the clinical data and information on the mutation events shared between the subclones with the same `matching_label` in the cluster trees. |

## References


Al Bakir M, Huebner A, 3tínez Ruiz C, *et al.* The evolution of non-small cell lung cancer metastases in TRACERx. *Nature* **616**(7957):534–542, 2023.

Baciu-Drăgan MA, Beerenwinkel N. Oncotree2vec - a method for embedding and clustering of tumor mutation trees. *Bioinformatics* **40**(Supplement_1):i180–i188, 2024.

Cannon M, Stevenson J, Stahl K, *et al.* DGIdb 5.0: rebuilding the drug-gene interaction database for precision medicine and drug discovery platforms. *Nucleic Acids Research* **52**(D1):D1227–D1235, 2024.

Caravagna G, Giarratano Y, Ramazzotti D, *et al.* Detecting repeated cancer evolution from multi-region tumor sequencing data. *Nature Methods* **15**(9):707–714, 2018.

Christensen S, Kim J, Chia N, *et al.* Detecting evolutionary patterns of cancers using consensus trees. *Bioinformatics* **36**(Suppl_2):i684–i691, 2020.

El-Kebir M, Satas G, Oesper L, *et al.* Inferring the mutational history of a tumor using multi-state perfect phylogeny mixtures. *Cell Systems* **3**(1):43–53, 2016.

Ellson J, Gansner E, Koutsofios L, *et al.* Graphviz—open source graph drawing tools. In P Mutzel, M Jünger, S Leipert, G Goos, J Hartmanis, van Leeuwen, editors, *Graph Drawing*, volume 2265 of *Lecture notes in computer science*, pages 483–484. Springer Berlin Heidelberg, Berlin, Heidelberg, 2002.

Grigoriadis K, Huebner A, Bunkum A, *et al.* CONIPHER: a computational framework for scalable phylogenetic reconstruction with error correction. *Nature Protocols* **19**(1):159–183, 2024.

Jahn K, Kuipers J, Beerenwinkel N. Tree inference for single-cell data. *Genome Biology* **17**:86, 2016.

Jamal-Hanjani M, Wilson GA, McGranahan N, *et al.* Tracking the evolution of non-small-cell lung cancer. *The New England Journal of Medicine* **376**(22):2109–2121, 2017.

Kuipers J, Tuncel MA, Ferreira P, *et al.* Single-cell copy number calling and event history reconstruction. *Bioinformatics* **41**(3), 2025.

Luo XG, Kuipers J, Beerenwinkel N. Joint inference of exclusivity patterns and recurrent trajectories from tumor mutation trees. *Nature Communications* **14**(1):3676, 2023.

Morita K, Wang F, Jahn K, *et al.* Clonal evolution of acute myeloid leukemia revealed by high-throughput single-cell genomics. *Nat Comm* **11**(1):5327, 2020.

Noble R, Burri D, Le Sueur C, *et al.* Spatial structure governs the mode of tumour evolution. *Nature Ecology & Evolution* **6**(2):207–217, 2022.

Razavi P, Chang MT, Xu G, *et al.* The genomic landscape of endocrine-resistant advanced breast cancers. *Cancer Cell* **34**(3):427–438.e6, 2018.

Sollier E, Kuipers J, Takahashi K, *et al.* COMPASS: joint copy number and mutation phylogeny reconstruction from amplicon single-cell sequencing data. *Nature Communications* **14**(1):4921, 2023.

Wegmann R, Bonilla X, Casanova R, *et al.* Single-cell landscape of innate and acquired drug resistance in acute myeloid leukemia. *Nature Communications* **15**(1):9402, 2024.
